# Combining remote sensing and cell-phone users’ mobility data to monitor the impact of transportation on NO_2_ concentrations in India*

**DOI:** 10.1088/2515-7620/ae654d

**Published:** 2026-05-07

**Authors:** Corbett Grainger, Adam Theising, Fan Zhang

**Affiliations:** 1 University of Wisconsin—Madison, and CESifo, Munich, Germany; 2 National Center for Environmental Economics, EPA, Munich, Germany; 3 World Bank, Munich, Germany

**Keywords:** pollution source attribution, cell phone mobility data, remote sensing, India

## Abstract

Estimating the extent to which transportation contributes to air pollution levels has been hampered by the difficulty in separating the relative degree of ambient NO_2_ generated by transportation, power generation, and industrial activity—all of which play roles. This paper addresses this gap by isolating the impact of ground-level mobility on air pollution in India through a combination of remotely sensed tropospheric NO_2_ measures and data from mobile-phone users’ locations. We construct vectors of ground-level movement of cell phones to estimate the impact of daily changes in mobility within a given district, controlling for both daily thermal electricity generation from upwind power plants and for trends in ambient pollution concentrations over time and space. We find that tropospheric NO_2_ concentrations are very responsive to changes in mobility, and that the effect varies with population density. In the most densely-populated regions, our findings show that a 1% increase in mobility increases NO_2_ concentrations by more than 2%, suggesting that traffic congestion plays a significant role in air pollution.

## 1. Introduction

The ability to accurately monitor emissions from transport-related activities with a high degree of spatial and temporal resolution is important for understanding the impact of transportation on air quality and human health. However, measuring the impact of traffic on ambient air pollution concentrations is difficult, particularly over large areas. The impact of traffic on pollution varies with vehicle characteristics, traffic congestion, and environmental conditions. There is a dearth of information on the number of vehicles on the road at any time. Even indicators of fuel use at the regional level are typically unavailable at the daily level. Traditional environmental indicators also have drawbacks; they are generally backward looking and often only available at coarse levels of spatial resolution. In developing country contexts, in particular, the collection of key environmental indicators relies on administrative procedures that take time and may be subject to biases resulting from existing political incentive structures (Sandefur and Glassman 2015).

In this paper, we use data from mobile-phone users’ locations to construct vectors of ground-level movement and combine these data with remotely sensed NO_2_ measures to better understand how transportation infrastructure usage impacts air pollution in India. To better measure the relative contribution of traffic to emissions, we exploit changes in mobility within a spatial unit to estimate elasticities of NO_2_ with respect to on-the-ground mobility. To understand the impact of ground-level transportation on pollution, our specifications leverage variations in mobility within a given district and by the day of the week. We control for daily thermal electricity generation by upwind power plants and use a combinations of fixed effects to condition on trends in ambient pollution concentrations over time and space.

We examine the impact of movement between 16 × 16 km pixels on a national grid, leveraging within-pixel-by-day-of-the-week variation in mobility. We find an average elasticity for ‘movement’ of greater than two, meaning that a 1% increase in movement between these tiles translates to more than a 2% increase in tropospheric NO_2_. We also find that the elasticity with respect to *non-movement* is asymmetrically larger in magnitude and negative. Our estimates suggest that a 1% increase in the number of people *who do not move* between tiles is associated with a decrease in NO_2_ concentrations of 3%–4%. In theory, we would expect an increase in the number of people *not moving* between tiles to offset the effect of people moving, but we highlight two measurement issues: (1) censoring, by which we mean the underreporting of movements; and (2) omitted variables bias, by which we mean that non-movement during the period we study was correlated with pandemic-related economic shutdowns, which affected other pollution sources. To minimize the impact of these issues, we use subsamples of the data from regions where censoring was less prominent, and from time periods pandemic-related lockdown policies were not in effect in India.

This paper contributes to several strands of literature. First, the mechanisms through which traffic impacts emissions and pollution concentrations are of broad interest, particularly in developing-country contexts. Studies in this line of the literature have thus far tended to focus on single urban environments, such as Seattle (Xiang *et al* 2020) and the Danish cities of Copenhagen and Roskilde (Khan *et al* 2020); Fu *et al* (2020) simulate the dispersion of NO_*x*_ from traffic emissions in Baoding, Hebei, China. Misra *et al* (2013) provide an example of an integrated-modeling approach to understand the impact of urban traffic emissions in Canada. (See Colvile *et al* (2001) for an overview of this literature.)

Second, this paper contributes to the literature that studies the impacts of various policy changes on commuting behavior. Infrastructure and public transportation affect commuter behavior and, thus, pollution. Recent empirical work shows that the density of subway-network coverage has a significant impact on pollution in China (Li *et al* 2019), and that expansions in regional train service in Germany reduced carbon monoxide and nitrogen dioxide (Lalive *et al* 2018). Tolls and congestion charges offer alternative policies to combat pollution from traffic because an increase in the cost of driving should induce substitution toward public transit or change the timing of trips during the day. The literature documents that temporary bans on car trips reduced pollution in Santiago, Chile (Rivera 2021), and that temporary driving restrictions in China led to a significant reduction in key criteria pollutants (Han *et al* 2020)^4^. Leveraging the waiver of tolls during a National Day holiday in 2012 in China, Fu and Gu (2017) find that the waiver led to pronounced increases in pollution.

Third, the paper contributes to recent studies in economics that have leveraged changes in pollution from transportation to study health and mortality outcomes. For example, Anderson (2020) shows that changes in wind direction from highway traffic impact mortality rates in Los Angeles. Knittel *et al* (2016) use an instrumental variable strategy to study the effect of particulate matter from traffic congestion on infant mortality and health in California, finding large effects on infant mortality. Currie and Walker (2011) leverage changes in congestion in New Jersey and Pennsylvania from the rollout of an electronic toll collection system (EZ-Pass) to study the impact of transportation emissions on infant health, also finding significant impacts. Zhou *et al* (2010) study the impact of driving restrictions on pollution in the lead-up to the 2008 Olympics in Beijing, documenting steep reductions of CO and NO_*x*_ emissions that result. Recent studies have also leveraged the COVID-19 lockdown policies to examine effects on ambient pollution (e.g. Cole *et al* 2020), but these studies measure the net impacts of a lockdown on air pollution, not the relationship between mobility and pollution.

Fourth, the paper adds to the increasing number of studies that use remote-sensing data to estimate ambient pollution concentrations with high spatial resolution (Hopkins *et al* 2016, Jiang *et al* 2016, Demetillo *et al* 2020, Shen *et al* 2021), or to show the long-term patterns of air pollution over a large geographic area (Li 2020). Akbar *et al* (2018) provide a useful descriptive example of *city-level* patterns of mobility and congestion from India. While there are descriptive studies of pollution patterns over time and space, few studies incorporate mobility or traffic data on a large scale. To the best of our knowledge, we are the first to isolate the impact of ground-level mobility on air pollution.

We proceed as follows: section 2 describes the data used in the analysis. Section 3 introduces our empirical approach and discusses the results. Section 4 offers conclusions and suggestions for future work.

## 2. Data description

NO_2_ is an emissions byproduct of combustion from cars, trucks, power plants, and industrial facilities. We are interested in tropospheric NO_2_, which is sensed remotely by several satellites maintained by the National Aeronautics and Space Administration (NASA) and the European Space Agency. We rely on NASA’s AURA ozone monitoring instrument (OMI) Level-3 product; this has a spatial resolution of roughly 25 × 25 km—a single *pixel* is the spatial unit of observation for our analysis—and it records daily observations of tropospheric vertical column densities (Krotkov *et al* 2019). The satellite passes over the Indian subcontinent in the early afternoon, giving daytime estimates of NO_2_ molecules in each tropospheric column—the area extending from Earth’s surface up to around 10 km. This product was selected primarily for computational ease at the geographic scale we study; pre-processing has been completed by NASA and the resulting pixel-cell size is relatively compact.

Pixel-level satellite observations are often missing, or they are flagged as unusable due to cloud cover, mechanical obstructions, or variations in satellite orbital paths (Duncan *et al* 2014). Following best practices and in an appeal to parsimony, we address the missing observations by relying on a backwards-looking, 14 d moving average taken at the pixel level, as shown in figure 1. This adaptation smooths noise due to missing observations, while allowing us to maintain an analysis at the daily level. In appendix B, we also present analysis using day-of, contemporaneous measurements, after taking care to remove flagged or missing observations. The sample of daily measurements used in our analysis spans from March 2020 through April 2021.

For our mobility data, we rely on Movement Maps (MM) provided by Meta (formerly Facebook). These are particularly well suited to our analysis. The data track mobility of users across the universe of Meta’s suite of mobile-phone apps (Facebook, Messenger, Instagram, WhatsApp)^5^.

Meta’s raw MM data compile aggregate counts of cell-phone movements between 16 × 16 km Bing tiles; therefore the data’s spatial unit of observation is a *tile pair*. We restrict the data sample to include only Bing tiles that fall within India’s national borders. The mobility data provide counts of movement between tile pairs (*i,j*) every 8 h. The starting (origin) and ending (destination) tiles are determined by the location of an individual’s mobile phone at the beginning and ending windows of time. The resulting movement matrix is analogous to a source-receptor matrix, with counts of people moving between all pairs (*i,j*).

For a given starting tile, we observe the daily count of movement flows to every other Bing tile that occurred in the eight-hour window between 5:30am (0:00 UTC) and 1:30pm (8:00 UTC). This afternoon cutoff timing coincides with the OMI satellite’s daily NO_2_ readings at 1:30pm local time. Because NO_2_ has a short photochemical lifetime—typically between 2 and 20 h, with variation in ambient lifetime largely attributable to seasonality and concentration levels of NO_*x*_ in the atmosphere (Beirle *et al* 2011, Shah *et al* 2020)—we can link morning inter-tile population movements with contemporaneous tropospheric nitrogen dioxide levels, as measured at the end of the movement period. Moreover, the relatively fine spatial dimensions of the MM data permit us to map mobility statistics onto individual NO_2_ pixel cells and to conduct analysis at that level. Most publicly available mobility datasets (e.g. Google Community Mobility, Apple Mobility Trends) provide only summary data at far larger spatial scales, such as the district or metropolitan-area levels.

To link our mobility and NO_2_ measures, we first map all origin and destination mobility tiles to their spatially-intersecting pollution pixel(s). We then aggregate all movement counts up to the pixel level, creating movement flows at the same resolution of the NO_2_ data. In cases where mobility tiles and pollution pixels do not line up perfectly, we assign movement proportionally based on overlapping area^6^. Having aggregated both sets of spatial data to a consistent level, we henceforth refer to the unit of observation in this analysis as a ‘pixel’. The primary mobility measures we generate for our analysis are simple in nature, and we calculate the measurements for approximately 4000 pixels each day). The first measure is the sum of all *non-movers*, which we define as those people in a given NO_2_ pixel cell whose mobile phones remained in the same NO_2_ pixel at the beginning and ending times of day that we examine. The second measure is a count of all *movers* associated with a pixel; this includes any phone that starts in one Bing tilebut ends in another. This measure is a rough daily proxy for total population mobility. To conduct heterogeneity analyses, we also decompose this movement measure by distance. We separately sum mover counts by distance traveled: 0–20 km, 20–50 km, 50–100 km, or greater than 100 km.

The mobility data come with several limitations that warrant a brief discussion. First, due to the construction of the dataset, we can only count individuals moving *between* pixels; an individual may be moving about *within* a pixel during the day, but that individual would be measured as a non-mover. We note that this movement would be at most roughly 23 km. Our specifications include spatial-unit fixed effects (or interactions with day-of-week indicators), so, to the extent that this movement is constant or cyclical within a week, it will be absorbed by our regressions’ fixed effects.

Second, in some cases, the data are censored^7^ this censoring leads to an underestimate of movement in certain pixel pairs, including those that are over long distances and/or are in very rural areas. Though this bias is likely smaller in urban areas where population densities are higher, there are more people moving between pixel pairs than our estimates would indicate.

Third, developing a quantitative understanding of the representativeness of mobility data generated from smartphone use remains an active area of academic research. Though Meta’s family of social media applications are among the most widely used in India, user demographics skew towards middle- or high-income and early- or middle-age. Moreover, the mobility data is based on movements of users who elect to share their location through app usage. Some research has found that biases in representation from differential phone ownership rates and differential app use patterns have only a limited bias effect on measures of total population mobility, even in lower-income countries (Wesolowski *et al* 2013, Ilin *et al* 2021); others have found meaningful degrees of bias in mobility measures generated from such data (Milusheva *et al* 2021). We therefore only claim our analysis as representative of movement by Indian smartphone users and acknowledge that we have no way to independently assess the national representativeness of these mobility data.

We turn finally to a brief description of our point source pollution data. We use a rich set of electricity generation data for the entirety of India. Because NO_2_ emissions are produced both *directly* by coal- and gas-powered electricity generation and *indirectly* through industrial processes that also use this generation, we use these data primarily to control for non-transportation-related nitrogen oxides in the air^8^. In their raw form, our electricity data provide daily total megawatts generated during our study period by 533 facilities across the country. See figure 2 for an overview of these facilities’ locations^9^.

Because NO_2_ emissions from a power plant can travel dozens of kilometers away from their point source, it is important to account for potential pollution spillovers over space. We control for changes in wind patterns for each thermal generator, relating each electricity generation point source to a set of downwind NO_2_ -pixel-cells. We use time-varying information on predominant wind directions to calculate a monthly downwind buffer area with radius 100 km for each power plant stack location^10^. We make a commonly-used simplifying assumption that ‘downwind’ areas can be characterized as a quadrant (i.e. a 90° slice) centered on the predominant monthly wind direction away from the plant. For example, figure 3(a) shows the average prevailing wind for January 2020, and figure 3(b) illustrates the direction for June 2020. Using these monthly facility-level downwind buffer areas, we spatially join facilities to their downwind NO_2_ -pixel-cells. Then for each NO_2_ -pixel-cell, we calculate the aggregate daily generation at all (month-specific) upwind thermal power facilities. While this assumption does not capture the full complexity of pollutant transport and dispersion, a multidisciplinary body of research—including in multiple studies in India—has found that coal power generation affects downwind ambient pollution concentrations out to distances of 100 km (Barrows *et al* 2019, Clay *et al* 2024, Tanaka 2024, Singh *et al* 2025). In our regression analyses we will find a similar correlation between downwind NO_2_ and upwind levels of coal generation.

We conclude by overlaying these datasets in a single illustrative visualization. In figure 4, we highlight each key component of our data on 16 April 2020. The map is situated over northern India; it includes the cities of New Delhi and Chandigarh, as well the neighboring states of Haryana and eastern Punjab. The gridded background coloring illustrates the NO_2_ pixel-cell raster. Relatively higher NO_2_ concentrations are shown in yellow, while lower concentrations are shown in purple/red. The black diamond points are power plant locations, from which we draw measures of upwind thermal generation. The orange-to-red points and lines visualize a selected subset of the movement data. To minimize visual clutter, we illustrate these data for three origin tiles: one each in New Delhi, Chandigarh, and Haryana. These are marked with the red squares.

For each of these three origins, flowlines are drawn to all local destinations (<100 km) towards which we observe at least ten movements during morning hours. To simplify interpretability, the color of the lines between origin and destination differentiate mobility percentage declines relative to a baseline established in January and February 2020^11^. Darker red lines show a relatively soft decline during this early period of the COVID-19 pandemic, while the brightest orange lines denote that mobility fell to nearly 20% of baseline levels on this date.

## 3. Empirical analysis

Our goal is to identify the impact of ground-level transportation on ambient NO_2_ concentrations. We leverage variation in electricity demand and mobility due to COVID-19-related restrictions during 2020–21 and use fixed effects to obtain ‘within-pixel’ or ‘within-pixel-by-day-of-week’ variation over time. We define an observation at the pixel-by-day level, where a pixel is defined by the gridded NO_2_ data.

### 3.1. Empirical approach

We are interested in estimating the impact of transportation on atmospheric concentrations of NO_2_ using pixel-to-pixel movements as a proxy for the level of transportation activities. We also control for the number of individuals *not* moving between pixels in a day. Our baseline regression specification is represented by

$$\text{arsinh}\left({\text{NO}}_{2\text{idt}}\right)=\alpha_{1}\text{arsinh}\left(\left.{\text{Generation}}_{\text{it}}\right)+b_{1}\text{arsinh}\left(MM_{\text{it}}\right)+b_{2}\text{arsinh}H\left(NM_{\text{it}}\right)+\gamma_{\text{id}}+\varepsilon_{\text{ist}},\right.$$

where arsinh(NO_2*ist*_) is the inverse hyperbolic sine of remotely sensed NO_2_ in pixel *i*, day of the week *d* and date *t*. We choose this transformation because it has similar properties as a natural logarithm but is defined at zero. The instantaenous elasticity of NO_2_ with respect to mobility is $\varepsilon =\frac{\partial \text{ln}\left({\text{NO}}_{2}\right)}{\partial \text{ln}(\text{mobility})}=\beta \frac{\text{mobility}}{\sqrt{1+{\text{mobility}}^{2}}}\frac{\sqrt{1+{\left({\text{NO}}_{2}\right)}^{2}}}{{\text{NO}}_{2}}$. For large values of the variables of interest, the coefficients can be interpreted directly as elasticities (see Bellemare and Wichman (2020) for a discussion).

On the right-hand side of the equation, *Generation*_*it*_ is electricity generation upwind of pixel *i* on date *t*, while *MM* represents the numbers of people moving and *NM* represents those not moving across pixels in the data. Finally, *γ*_*id*_ represents a vector of pixel-by-day-of-week fixed effects, and *ε*_*ist*_ is an idiosyncratic error term. Because the error term may exhibit residual spatial and temporal autocorrelation, we estimate standard errors that are clustered by month-year and by administrative district (Cameron *et al* 2011).

Our preferred specifications utilize day-of-week-by-pixel fixed effects. This allows us to control non-parametrically for pixel-specific fluctuations in pollution throughout the week; it also ensures that we are isolating within-pixel variation in mobility, thermal generation, and NO_2_. Our focus is on estimating the partial relationship between mobility and NO_2_ using within-cell, within-time variation; accordingly, overall goodness-of-fit measures (e.g. adjusted *R*^2^) are not expected to be high. Descriptive statistics for the key variables are shown in table 1. To highlight the role of censoring in the mobility data we also show the proportion of observations that are censored and the conditional mean (i.e. the mean conditional on there being at least 10 individuals observed). Variation in mobility by state is illustrated in Figure 5.

We note that satellite NO_2_ retrievals are noisy at the daily cell level. In our setting NO_2_ is the dependent variable, so under the standard assumption that retrieval error is mean-zero conditional on the included fixed effects and controls, this measurement error does not generate attenuation bias in the estimated response of NO_2_ to mobility; it increases the variance of the regression residual and reduces precision. Our main specifications use a moving average of NO_2_, which smooths out daily errors, but we also show all specifications in appendix B utilizing daily pixel-level estimates.

The average number of individuals in a pixel who do not leave the pixel on a given day is 5168, with a standard deviation nearly 4 times the mean. Figure 4 illustrates these differences in mobility across the states of India.

The baseline fixed-effects regression results are shown in tables 2 and 3. As expected, the estimated elasticities vary with the level of fixed effects included in the estimation. In table 2 results are shown for the impact of mobility on NO_2_. In column (1) of table 2, the implied elasticity using the formula above implies that a 10% increase in mobility leads to a 0.98% increase in NO_2_ (i.e. the elasticity is slightly smaller than interpreting the coefficient directly).

By contrast, in table 3 column (1), a 10% increase in *non-movement*, holding all else constant, leads to a *decrease* in NO_2_ concentrations of 23%. For ease of interpretation, we will treat coefficients as approximate elasticities in the remainder of the discussion. Appendix B shows elasticities directly for alternative specifications.

As shown in table 1, censoring is a concern in the mobility measures. The movement between pixels *i* and *j* is not reported if there are fewer than 10 individuals observed. We are also concerned with mobility being correlated with changes in economic activity driven by the pandemic and related policies. To address these concerns, we now estimate an analogous model for a subsample of the observations without zeros (i.e. where some movement is detected, but where movement between pixel pairs may still be unreported). We also restrict the analysis to include observations from June to November 2020, a period when economic activity had resumed and the Delta variant of COVID-19 had not been identified as dominant in India.

Tables 4 and 5 show the results for *movement* and *non-movement*, respectively. The preferred specifications for mobility indicate that the impact of movement on NO_2_ is more than unit elastic; a 1% increase in movement leads to roughly a 2.5% increase in NO_2_. *Non-movement* has a larger impact in absolute value; a 1% increase in the number of individuals staying home, relative to the mean for that pixel, leads to a 4% decrease in NO_2_.

### 3.2. Heterogeneity between states

There is significant heterogeneity in NO_2_ levels, movement, and power generation between states. The specifications so far have restricted the elasticities of NO_2_ with respect to mobility to be the same across states. To test for heterogeneous responses of pollution concentrations to mobility we estimate the regressions separately by state.

Table 6 shows the results for the three most populous states in India: Uttar Pradesh, Maharashtra and Bihar. As before, we separately estimate the elasticities with respect to total movement and total *non-movement*. The results are largest in magnitude for Maharashtra, with estimated elasticities of 7.5 for movement and −9.7 for non-movement. These estimates suggest that congestion in this highly urbanized state contributes significantly to NO_2_.

### 3.3. Robustness and other evidence

In addition to the fixed-effects approach in tables 2–6, we also use a two-step approach, in which we first estimate a model of NO_2_ concentrations as a function of power generation and a suite of fixed effects and local trends for data from 2014 through March 2020. We use the model to predict (out of sample) NO_2_ concentrations in 2020 and 2021, and we then use the residual as a new dependent variable that is regressed on measures of mobility. This allows us to leverage the longer panel structure of the power generation and NO_2_ data. The results, shown in appendix A, further support our findings in this paper.

Finally, in addition to our main specifications in tables 2–6, we also estimate the same models with contemporaneous daily NO_2_ as the measure of interest in appendix B. The results are robust to these specifications as well as alternatives moving averages (e.g. 4 d, 7 d moving average).

## 4. Conclusions

Fossil fuel combustion from transportation is known to cause air pollution, but separately identifying the impact of transportation on NO_2_ concentrations has been difficult due to data limitations and confounding factors. Measuring the impact of traffic on ambient air pollution concentrations is difficult, particularly over large areas, because the impact of traffic on pollution varies with vehicle characteristics, traffic congestion, and environmental conditions. There is also a dearth of information on the number of vehicles on the road at any time, and for daily indicators of fuel use at regional levels. We overcome these issues by combining remote-sensing estimates of tropospheric NO_2_ in India with data on electricity generation and on the movement of mobile phones. This combination allows us to estimate the responsiveness of tropospheric NO_2_ to population movements.

We find that ground-level mobility has a large, significant impact on NO_2_ concentrations. The average elasticity for ‘movement’ estimated nationwide is roughly 2.5, meaning that a 1% increase in movement translates to a 2.5% increase in tropospheric NO_2_. We also demonstrate that there is significant heterogeneity over space. In Maharashtra, for example, the estimated elasticities can be two to three times the average elasticity. Maharashtra is relatively industrialized and has higher incomes than most of India, which could be a driver in this difference.

We also find that NO_2_ concentrations are very responsive to within-pixel changes in the number of individuals *not moving long distances during the day*. Because this measure is less contaminated by censoring, the elasticity is larger in magnitude. Our preferred estimates suggest that a 1% increase in the number of individuals *not traveling long distances* decreases NO_2_ concentrations by about 4%.

We emphasize that our findings must be interpreted with several caveats related to the data products on which our analysis is built. While remotely sensed measures of NO_2_ and other air pollutants have proven invaluable to researchers studying pollution in lower- or middle-income countries, there remains structural uncertainty on the order of 30%–50% in tropospheric NO_2_ retrievals from OMI (Boersma *et al* 2018). Furthermore, our use of a NO_2_ data product produced by a polar-orbiting satellite with a single early afternoon overpass may not fully capture diurnal variability in NO_2_ that is meaningfully correlated with transportation-related emissions (Penn and Holloway 2020). We noted several caveats related to our mobility data in section 2, but remphasize here that we have not quantitatively assessed whether the Meta userbase’s movement patterns are nationally representative or only reflective of a selective subpopulation And finally, we have taken a very parsimonious approach to controlling for upwind NO_2_ emissions from power plants, but note that we lack data on capture diesel generation. In the case where (unobservable) local captive generation levels are correlated with movement patterns but *not* with upwind power plant generation levels, there is potential for omitted variable bias.

There are several areas for future research. First, machine learning could help researchers use the rich data available to better understand how the effects vary with other covariates, such as weather, topography, and land use. One could also estimate the effects separately for smaller spatial units, such as neighborhoods or cities, as well as determining how the effects vary with demographic characteristics. Future work may be able to precisely estimate movement elasticities over these smaller areas or shorter time periods by applying advanced computational methods to remote sensing monitoring products with more granular spatial resolution (e.g. TROPOMI, which measures NO_2_ on a 3.5 × 5.5 km grid) or those derived from geostationary instruments (e.g. GEMS, TEMPO, Sentinel-4) that permit measurement throughout the day (Zoogman *et al* 2017, Kim *et al* 2020). Finally, we note that our findings are for NO_2_ concentrations, but further work should be done to determine the impact on other pollutants as well as greenhouse gas emissions.

## Figures and Tables

**Figure 1.**
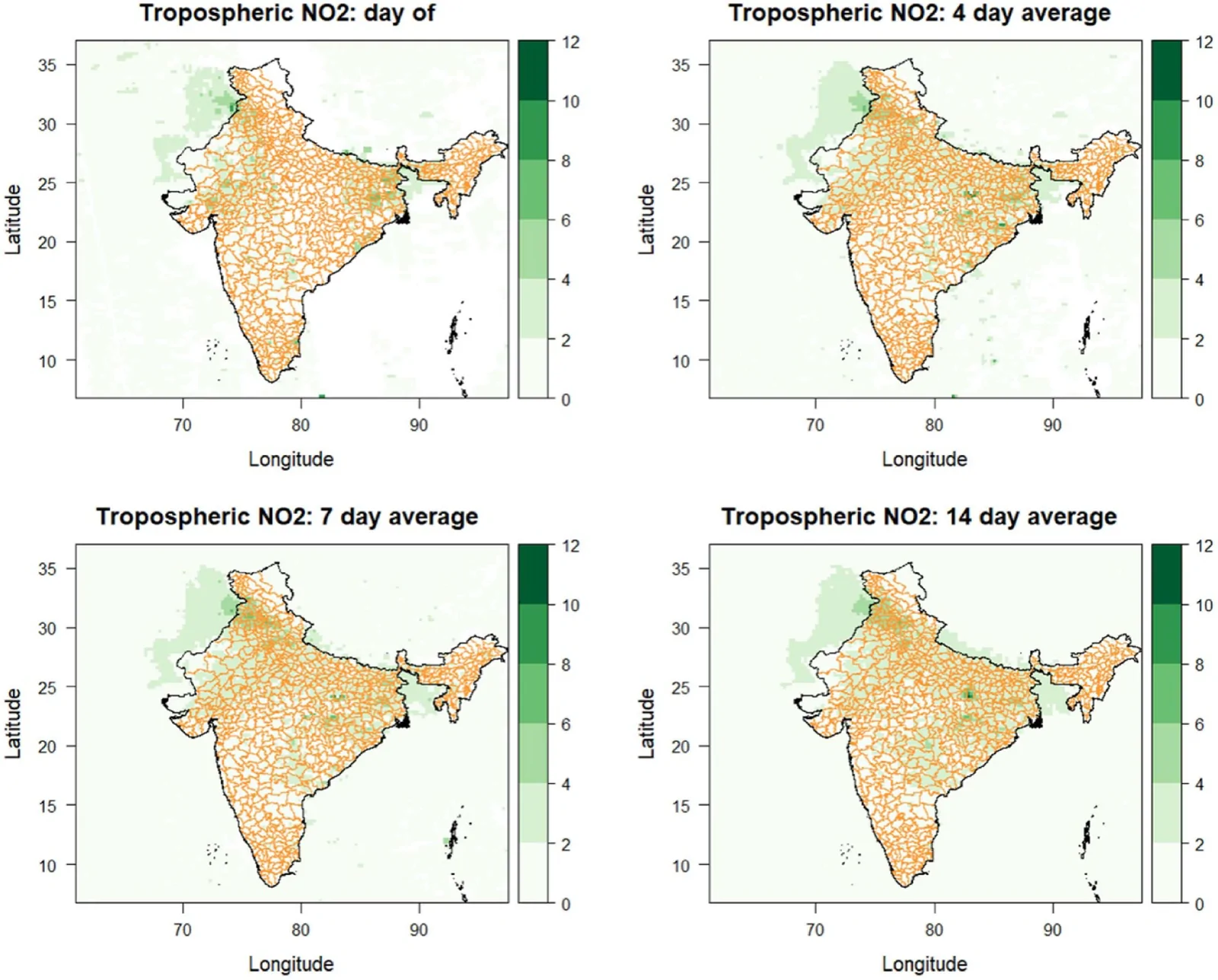
Comparisons of raw and cleaned daily NO_2_ estimates.

**Figure 2.**
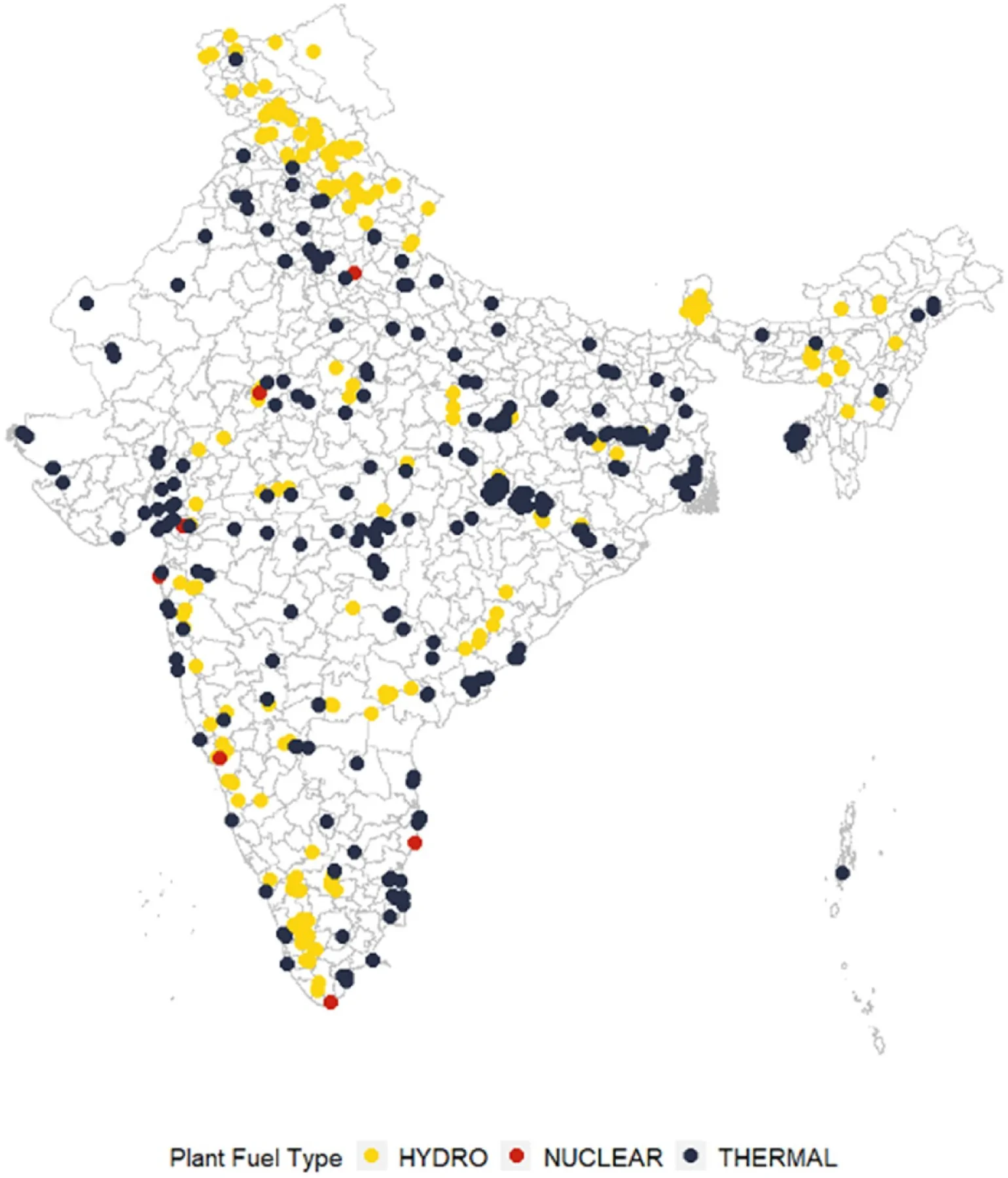
Location of Indian power plants, by fuel type.

**Figure 3.**
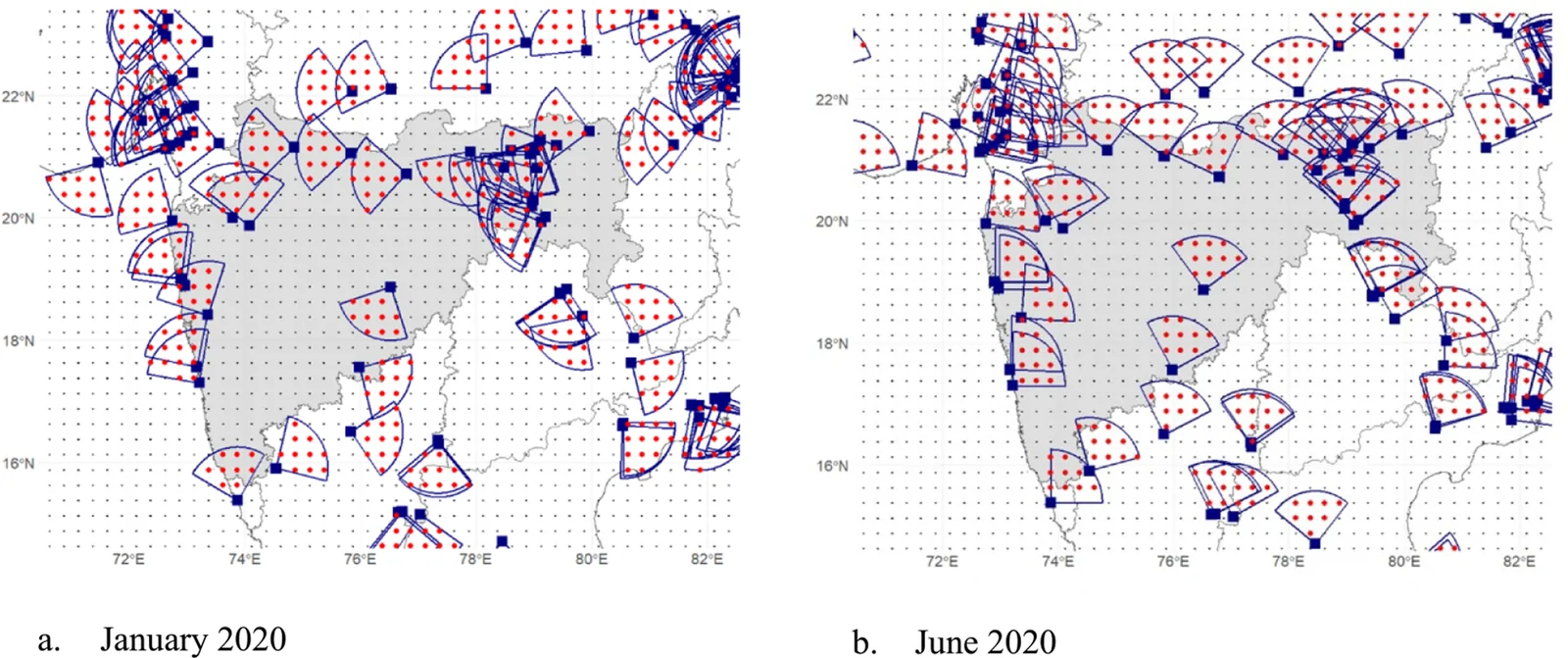
Defining downwind regions for thermal power plants.

**Figure 4.**
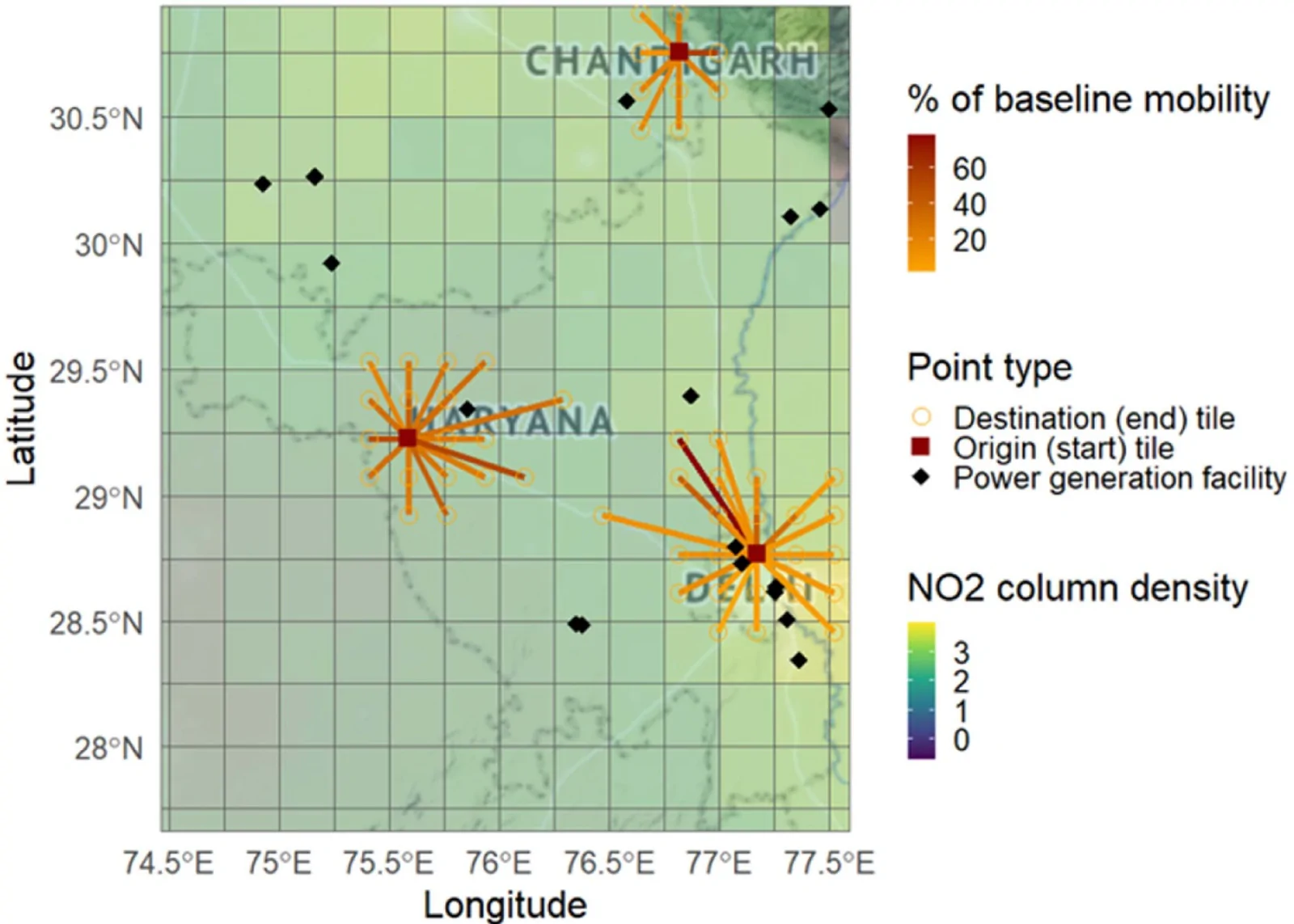
Overlaying NO_2_ data with plant-level generation and granular mobility data.

**Figure 5.**
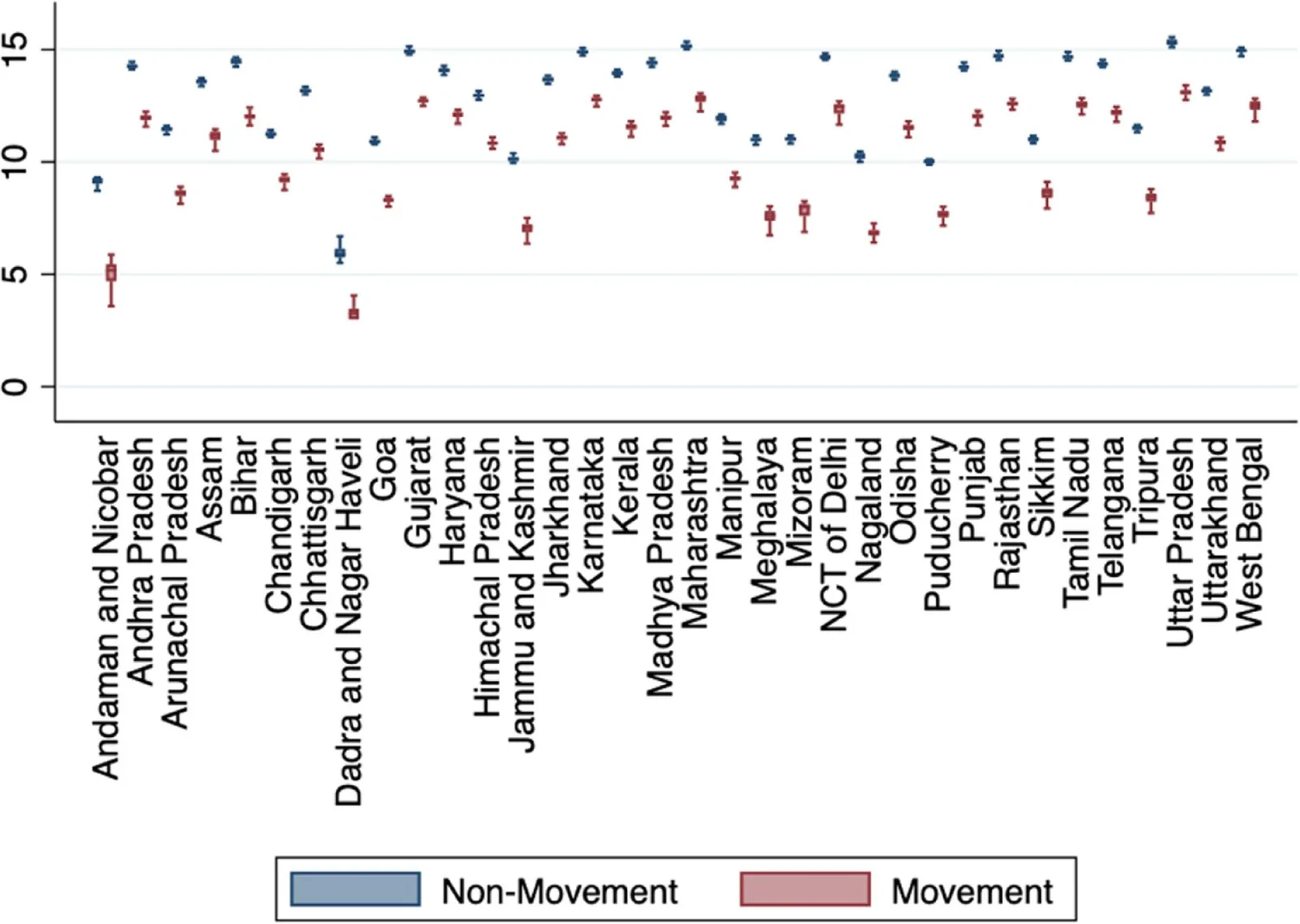
Variation in mobility and non-mobility counts across states.

**Table 1.** NO_2_ pixel-level means and standard deviations.

	Mean (Std dev)	Proportion censored	Conditional mean (uncensored observations)
NO_2_ (Remotely sensed) 14 d avg	2.11e + 15 (1.37e + 15)		
NO_2_ (Remotely sensed) daily*	2.23e ± 15 (1.85e ± 15)		
Daily electricity generation	0.6412 (5.6998)		
Upwind Elec. Gen. within 100 km	18.7066 (36.1052)		
Individuals staying in same pixel	5167.513 (21 026.08)	0.000 001 35 (0.0012)	5167.513 (21 026.08)
Individual movement btw pixels 0–20 km	361.7216 (1391.589)	0.1408 (0.3478)	421.0031 (1492.961)
Individual Mvmt btw pixels 20–50 km	120.8068 (477.0327)	0.3370 (0.4727)	182.2031 (576.2153)
Individual Mvmt btw pixels 50–100 km	24.4723 (102.5978)	0.7002 (0.4582)	81.6179 (174.4774)
Individual Mvmt btw pixels >100 km	10.1020 (73.5048)	0.8863 (0.3174)	88.8743 (201.3282)

*Notes*: Authors’ calculations using the datasets described in this paper and including all pixel-level data from 2020–2021 (March 2020—April 2021). There are 1483 558 pixel-by-day observations. For the NO_2_ daily variable, there are 756 144 pixel-by-day observations. ‘Mvmt btw’ is abbreviation for ‘movement between’.

**Table 2.** Fixed-effects regressions, pixel level.

	(1)	(2)	(3)	(4)
arsinh(Total Mvmt btw pixels)	0.114** (0.0107)	0.134** (0.0116)		
arsinh(Mvmt btw pixels, 0–20 km)			0.105** (0.0110)	0.126** (0.0121)
arsinh(Mvmt btw pixels, 20–50 km)			0.0636** (0.00648)	0.0688** (0.00683)
arsinh(Mvmt btw pixels, 50–100 km)			0.0354** (0.00601)	0.0372** (0.00625)
arsinh(Mvmt btw pixels, >100 km)			0.0114 (0.00809)	0.0124 (0.00838)
arsinh(Upwind Gen < 100 km)	0.0259** (0.00491)	0.0258** (0.00495)	0.0260** (0.00491)	0.0258** (0.00495)
Pixel FE	X		X	
DOW FE	X		X	
Month-by-Year FE	X	X	X	X
Pixel-by-DOW FE		X		X
Adjusted *R*-squared	0.093	0.081	0.093	0.081

The dependent variable is arsinh(NO_2_). Columns (1)–(4) present results for alternative regression specifications. There are 1469 328 (pixel-by-day) observations).

* and ** represent significance at the 5% and 1% levels, respectively. The within-pixel standard deviation of ‘Total Movement’ is 0.534. ‘Mvmt btw’ is abbreviation for ‘movement between’. ‘Gen’ is abbreviating ‘generation’. ‘DOW’ is abbreviating day of week.

**Table 3.** Fixed-effects regressions, full sample, pixel level.

	(1)	(2)	(3)	(4)
arsinh(Total ‘Non-Movers’)	−2.371** (0.0330)	−0.0899 (0.0520)	−0.0913 (0.0523)	−0.0947 (0.0534)
arsinh(Upwind Thermal Gen < 100 km)	−0.0544** (0.00484)	0.0263** (0.00491)	0.0263** (0.00491)	0.0262** (0.00495)
Pixel FE	X	X	X	
DOW FE	X		X	
Month-by-Year FE		X	X	X
Pixel-by-DOW FE				X
Adjusted *R*-squared	0.073	0.093	0.093	0.081

The dependent variable is arsinh(NO_2_). Columns (1)–(4) present results for alternative regression specifications. There are 1469 328 (pixel-by-day) observations.

* and ** represent significance at the 5% and 1% levels, respectively. The within-pixel standard deviation of ‘Non-movers’ is 0.169. ‘Mvmt btw’ is abbreviation for ‘movement between’. ‘Gen’ is abbreviating ‘generation’. ‘DOW’ is abbreviating day of week.

**Table 4.** Pixel-level regressions: June–November 2020, dropping censored observations.

	(1)	(2)	(3)	(4)
arsinh(Total Mvmt btw pixels)	2.411** (0.204)	2.775** (0.223)		
arsinh(Mvmt btw pixels, 0–20 km)			0.199 (0.294)	0.244 (0.334)
arsinh(Mvmt btw pixels, 20–50 km)			0.364* (0.182)	0.323 (0.204)
arsinh(Mvmt btw pixels, 50–100 km)			0.548** (0.0907)	0.672** (0.0999)
arsinh(Mvmt btw pixels, >100 km)			0.365** (0.0714)	0.367** (0.0766)
arsinh(Upwind Gen < 100 km)	0.0246 (0.0275)	0.0213 (0.0283)	0.0236 (0.0274)	0.0198 (0.0283)
Pixel FE	X		X	
DOW FE	X		X	
Pixel-by-DOW FE		X		X
Adjusted *R*-squared	0.080	0.051	0.081	0.053

The dependent variable is arsinh(NO_2_). Columns (1)–(4) present results for alternative regression specifications. There are 63 475 (pixel-by-day) observations.

* and ** represent significance at the 5% and 1% levels, respectively. The sample is restricted to June–November 2020, and to observations without censoring. ‘Mvmt btw’ is abbreviation for ‘movement between’. ‘Gen’ is abbreviating ‘generation’. ‘DOW’ is abbreviating day of week.

**Table 5.** Pixel-level regressions: June–November 2020, dropping censored observations.

	(1)	(2)
arsinh(Total ‘Non-movers’)	−3.950** (0.0895)	−4.043** (0.0919)
arsinh(Upwind Thermal Gen < 100 km)	0.0228* (0.00949)	0.0225* (0.00962)
Pixel FE	X	
DOW FE	X	
Month-by-State		
Pixel-by-DOW FE		X
Adjusted *R*-squared	0.106	0.083

The dependent variable is arsinh(NO_2_). Columns (1)–(2) present results for alternative regression specifications. There are 732 066 (pixel-by-day) observations.

* and ** represent significance at the 5% and 1% levels, respectively. The sample is restricted to June–November 2020 and observations without censoring. ‘Mvmt btw’ is abbreviation for ‘movement between’. ‘Gen’ is abbreviating ‘generation’. ‘DOW’ is abbreviating day of week.

**Table 6.** State-specific estimates for three most populous states (pixel-level specification).

	(1)	(2)	(3)	(4)	(5)	(6)
	U. Pradesh	U. Pradesh	Maharashtra	Maharashtra	Bihar	Bihar
arsinh(Total Mvmt)	1.162** (0.214)		7.511** (0.860)		0.562 (0.313)	
arsinh(Total ‘Non-movers’)		−0.633** (0.106)		−9.6823** (0.3659)		−0.550** (0.163)
arsinh(Upwind Gen)	−0.00491 (0.0272)	−0.0615** (0.0121)	−0.384** (0.140)	0.0389** (0.0406)	0.0265 (0.0414)	0.0443** (0.0152)
Pixel-by-DOW FE	X	X	X	X	X	X
N	8464	64 394	5418	72 532	2893	24 839
Adjusted *R*-squared	−0.006	0.016	0.022	0.0589	0.018	0.018

The dependent variable is asinh(NO_2_). Columns (1)–(6) present results for alternative regression specifications. Observations are at the pixel-by-day level.

* and ** represent significance at the 5% and 1% levels, respectively. The sample is restricted to June–November 2020, and to observations without censoring. ‘Mvmt’ is abbreviation for ‘movement’. ‘Gen’ is abbreviating ‘generation’. ‘DOW’ is abbreviating day of week.

**Figure 1A.**
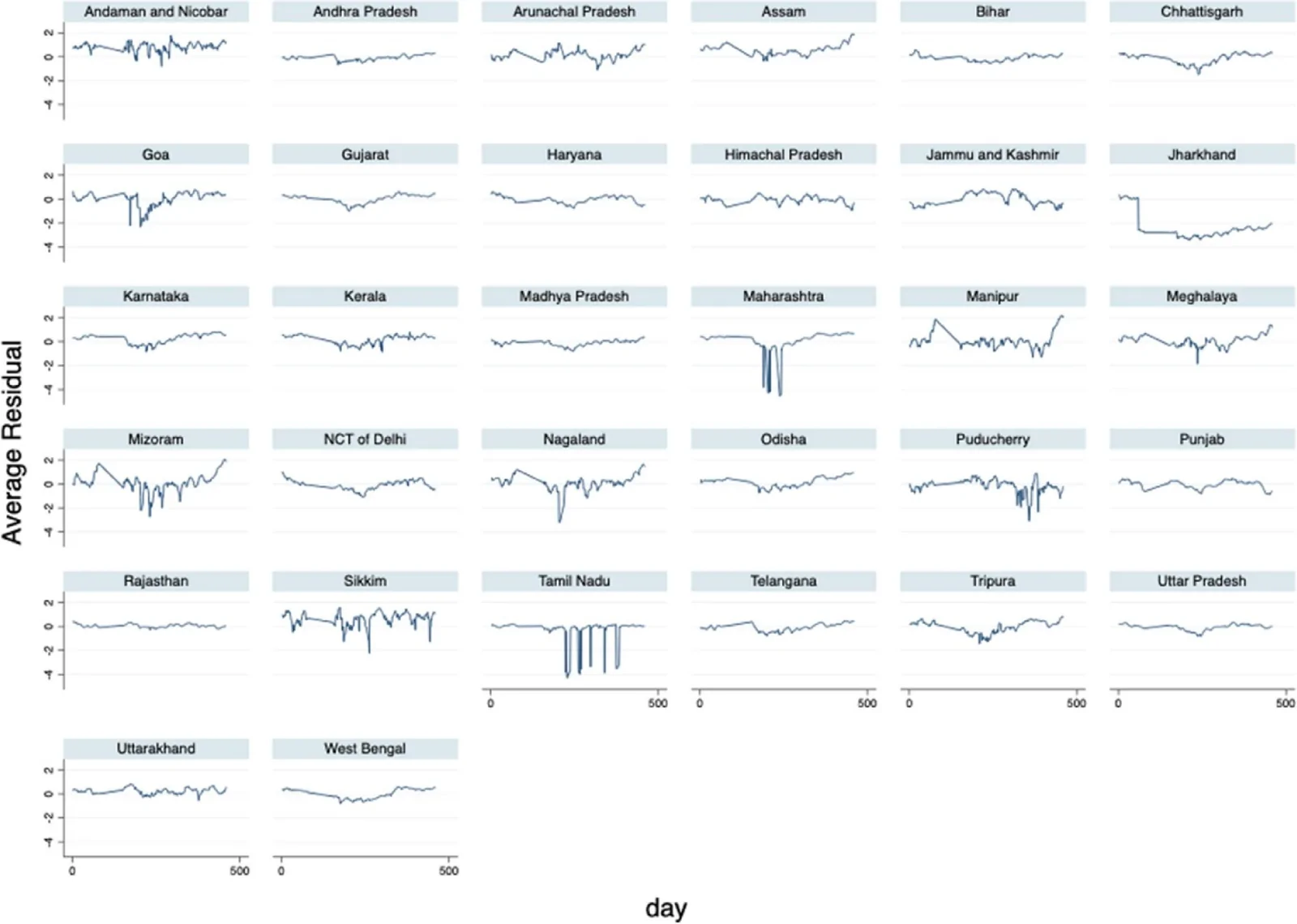
Average residuals by state and date. *Note*: The plots show average residuals at the state-by-day level. Residuals were calculated by taking the actual arsinh(NO_2_) levels minus out-of-sample predictions from model (3) of table 5. For graphical exposition some outliers were dropped from this plot (those with an absolute value of greater than five).

## Data Availability

The data cannot be made publicly available upon publication because they are owned by a third party and the terms of use prevent public distribution. The data that support the findings of this study are available upon reasonable request from the authors.

## Footnotes

4 There are also analytical models of urban environments that integrate traffic and pollution from mobile and point sources (e.g. Kyriakopoulou and Picard 2021).

5 Facebook, WhatsApp, Instagram, and Messenger were four of the five most widely adopted social media applications in India during the study period (see: https://datareportal.com/reports/digital-2021-india).

6 As a simplified example, suppose that two pollution pixels overlap with movement tile *i*. We assign the movement associated with tile *i* to the two pollution pixels based on the how much overlap there is with the two pollution pixels. If 75% of the area of tile *i* overlaps with the first pollution pixel, we assign 75% of the total movement of *i* to that pollution pixel and 25% to the other.

7 In the underlying MM data, if fewer than ten people move between a tile pair (*i,j*), that pair is coded as zero.

8 Many industrial facilities in India also have onsite captive generators to ease production constraints when there are blackouts or supply shortages. We have no simple way to measure use of these backups.

9 Captive generation is not reported, and so it is missing from our analysis. Because buying electricity from the grid is less expensive than captive generation, it is likely the case that there was little captive generation during the period of study because reported shortages were rare.

10 Wind direction data come from the fifth generation (ERA5) atmospheric reanalysis of climate data produced by the Copernicus Climate Change Service (3CS) at the European Centre for Medium-Range Weather Forecasts (ECMWF). See Hersbach *et al* (2019) for further details.

11 We do this for illustration purposes because population levels (and corresponding movement flows) vary by orders of magnitude across these three example origins, In our empirical specifications, however, we do not define movement (or non-movement) relative to a baseline; instead specifications rely on within-pixel deviations from the mean for that sample period.

## Appendix A. Leveraging a Longer Data Series

The results so far have been based on data beginning in 2020, when the mobility data first became available. In this subsection we leverage the longer panel of electricity generation and NO_2_ concentrations. We first estimate a high-dimensional fixed-effects model using data from 2013 to 2019. We include upwind power generation, day-of-week-by-pixel fixed effects, state-by-month fixed effects, and district-specific linear trends. Table 1A shows the results. The model explains a large share of the variation in NO_2_. We then use the model to predict out-of-sample NO_2_ concentrations in over the 2020–21 period, and the predictions are subtracted from the actual NO_2_ concentrations to generate residuals. The average residuals for each state are plotted over time in figure 1A. We then use the residuals at the pixel-by-day level as the dependent variable in a regression model, again restricting the sample to observations with reduced censoring as in tables 5 and 6. The results are shown in tables 1A and 2A. The results are similar in spirit, though with slightly smaller magnitudes.

**Table 1A.** Predictive model, pixel-level.

	
Electric generation	0.0217** (0.000616)
Gen. within 100 km	0.00976** (0.000185)
Gen. 100–200 km	0.00569** (0.000157)
Pixel-by-DOW FE	X
State-by-month FE	X
District-specific linear trend	X
Adj. *R*-sq	0.350

The dependent variable is arsinh(NO_2_). There are 9780 392 (pixel-by-day) observations.

* and ** represent significance at the 5% and 1% levels, respectively.

**Table 2A.** Explaining unexpected NO_2_ fluctuations: out-of-sample residuals (pixel level).

	(1)	(2)	(3)	(4)	(5)	(6)
arsinh(‘Non-movers’)	−1.684** (0.585)	−1.722** (0.612)				
arsinh(Total Mvmt)			0.147** (0.0478)	0.189** (0.0602)		
arsinh(Mvmt 0–20 km)					0.0927* (0.0399)	0.126* (0.0507)
arsinh(Mvmt 20–50 km)					0.0794** (0.0286)	0.0979** (0.0326)
arsinh(Mvmt 50–100 km)					0.0828 (0.0465)	0.0924 (0.0509)
arsinh(Mvmt >100 km)					0.0674 (0.0557)	0.0723 (0.0578)
Pixel FE	X		X		X	
Pixel-by-DOW FE		X		X		X
Adj. *R*-sq	0.997	0.997	0.997	0.997	0.997	0.997

The dependent variable is the residual from the predictive model described above. There are 732 067 (pixel-by-day) observations.

* and ** represent significance at the 5% and 1% levels, respectively.

## Appendix B

Tables 2B–6B correspond to the specfications in tables 2–6 using contemporaneous NO_2_ levels instead of the 14 d moving average. Our general findings are also robust to using a 4 d moving average or 7 d moving average; results available on request from authors.

**Table 2B.** Fixed-effects regressions, pixel level.

	(1)	(2)	(3)	(4)
arsinh(Total Mvmt btw pixels)	0.167** (0.0267)	0.171** (0.0290)		
arsinh(Mvmt btw pixels, 0–20 km)			0.100** (0.0275)	0.112** (0.0302)
arsinh(Mvmt btw pixels, 20–50 km)			0.112** (0.0159)	0.102** (0.0168)
arsinh(Mvmt btw pixels, 50–100 km)			0.0453** (0.0148)	0.0371* (0.0154)
arsinh(Mvmt btw pixels, >100 km)			0.0609** (0.0199)	0.0560** (0.0206)
arsinh(Upwind Gen < 100 km)	0.0505** (0.0125)	0.0543** (0.0127)	0.0512** (0.0125)	0.0548** (0.0127)
Pixel FE	X		X	
DOW FE	X		X	
Month-by-Year FE	X	X	X	X
Pixel-by-DOW FE		X		X
Elasticity of Row 1 Coefficient	0.138	0.142		
Adjusted *R*-squared	0.051	0.048	0.051	0.048

The dependent variable is arsinh(NO_2_). Columns (1)–(4) present results for alternative regression specifications. There are 756 134 (pixel-by-day) observations.

* and ** represent significance at the 5% and 1% levels, respectively.

**Table 3B.** Fixed-effects regressions, full sample, pixel level.

	(1)	(2)	(3)	(4)
arsinh(‘Non-Movers’)	−3.184** (0.0787)	−0.580** (0.129)	−0.602** (0.129)	−0.572** (0.132)
arsinh(Upwind Thermal Gen < 100 km)	−0.0513** (0.0122)	0.0541** (0.0125)	0.0521** (0.0125)	0.0558** (0.0127)
Pixel FE	X	X	X	
DOW FE	X		X	
Month-by-year FE		X	X	X
Pixel-by-DOW FE				X
Elasticity for ‘Non-Movers’	−2.984	−0.543	−0.564	−0.536
Adjusted *R*-squared	0.041	0.050	0.051	0.048

The dependent variable is arsinh(NO_2_). Columns (1)–(4) present results for alternative regression specifications. There are 756 134 (pixel-by-day) observations.

* and ** represent significance at the 5% and 1% levels, respectively.

**Table 4B.** Pixel-level regressions: June–November 2020, dropping censored observations.

	(1)	(2)	(3)	(4)
arsinh(Total Mvmt btw pixels)	3.726** (0.500)	4.260** (0.558)		
arsinh(Mvmt btw pixels, 0–20 km)			−1.186 (0.718)	−2.249** (0.836)
arsinh(Mvmt btw pixels 20–50 km)			0.980* (0.445)	1.531** (0.515)
arsinh(Mvmt btw pixels, 50–100 km)			0.645** (0.217)	0.823** (0.244)
arsinh(Mvmt btw pixels >100 km)			0.926** (0.168)	0.871** (0.184)
arsinh(Upwind Gen < 100 km)	0.0508 (0.0641)	0.0335 (0.0683)	0.0518 (0.0641)	0.0354 (0.0683)
Pixel FE	X		X	
DOW FE	X		X	
Pixel-by-DOW FE		X		X
Elasticity for total movement coefficient	3.495	3.996		
Adjusted *R*-squared	0.055	0.060	0.057	0.062

The dependent variable is arsinh(NO_2_). Columns (1)–(4) present results for alternative regression specifications. There are 28 376 (pixel-by-day) observations.

* and ** represent significance at the 5% and 1% levels, respectively. The sample is restricted to June–November 2020, and to observations without censoring.

**Table 5B.** Pixel-level regressions: June–November 2020, dropping censored observations.

	(1)	(2)
arsinh(‘Non-Movers’)	−6.679** (0.211)	−6.659** (0.218)
arsinh(Upwind Thermal Gen < 100 km)	0.0300 (0.0226)	0.0369 (0.0235)
Pixel FE	X	
DOW FE	X	
Month-by-State		
Pixel-by-DOW FE		X
Elasticity for Non-Movers	−6.100	−6.082
Adjusted *R*-squared	0.054	0.059

The dependent variable is arsinh(NO_2_). Columns (1)–(4) present results for alternative regression specifications. There are 319 026 (pixel-by-day) observations.

* and ** represent significance at the 5% and 1% levels, respectively. The sample is restricted to June–November 2020 and observations without censoring.

**Table 6B.** State-specific estimates for three most populous states (pixel-level specification).

	(1)	(2)	(3)	(4)	(5)	(6)
	U. Pradesh	U. Pradesh	Maharashtra	Maharashtra	Bihar	Bihar
arsinh(Total Mvmt)	2.071* (0.856)		11.41** (1.827)		1.432 (1.750)	
arsinh(‘Non-Movers’)		−4.663** (0.386)		−19.04** (0.881)		−4.061** (0.789)
arsinh(Upwind Gen)	−0.167 (0.109)	−0.0599 (0.0458)	−0.413 (0.299)	0.0203 (0.0949)	0.214 (0.216)	0.167* (0.0751)
Pixel-by-DOW FE	X	X	X	X	X	X
N	4118	31 103	2175	29 035	1223	10 210
Corresponding elasticity	2.025	−4.520	10.562	−16.914	1.383	−3.923
Adjusted *R*-squared	−0.020	0.001	0.020	0.029	−0.016	−0.000

The dependent variable is asinh(NO_2_). Columns (1)–(4) present results for alternative regression specifications. Observations are at the pixel-by-day level.

* and ** represent significance at the 5% and 1% levels, respectively. The sample is restricted to June–November 2020, and to observations without censoring.
